# Systematic evaluation of ^99m^Tc-tetrofosmin versus ^99m^Tc-sestamibi to study murine myocardial perfusion in small animal SPECT/CT

**DOI:** 10.1186/2191-219X-2-21

**Published:** 2012-05-24

**Authors:** Alexis Vrachimis, Sven Hermann, Domokos Máthé, Otmar Schober, Michael Schäfers

**Affiliations:** 1 European Institute for Molecular Imaging, University of Muenster, Mendelstrasse 11, Building L1, Muenster, 48149, Germany; 2 Department of Nuclear Medicine, University Hospital Muenster, Albert-Schweitzer-Campus 1, Building A1, Muenster, 48149, Germany; 3 Department of Biophysics and Radiation Biology, Faculty of Medicine, Nanobiotechnology and In Vivo Imaging Centre, Semmelweis University, Budapest, 1085, Hungary

**Keywords:** Multi-pinhole SPECT, Myocardial perfusion, MIBI, Tetrofosmin, Mouse

## Abstract

**Background:**

The “back-translation” of clinically available protocols to measure myocardial perfusion to preclinical imaging in mouse models of human disease is attractive for basic biomedical research. With respect to single-photon emission computed tomography (SPECT) approaches, clinical myocardial perfusion imaging protocols are established with different ^99m^Tc-labeled perfusion tracers; however, studies evaluating and optimizing protocols for these tracers in high-resolution pinhole SPECT in mice are lacking. This study aims at evaluating two clinically available ^99m^Tc-labeled myocardial perfusion tracers (^99m^Tc-sestamibi vs. ^99m^Tc-Tetrofosmin) in mice using four different imaging protocols.

**Methods:**

Adult C57BL/6 male mice were injected with ^99m^Tc-sestamibi (MIBI) or ^99m^Tc-Tetrofosmin (TETRO) (4 MBq/g body weight) either intravenously through the tail vein (*n* = 5) or retroorbitally (*n* = 5) or intraperitoneally (i.p.) under anesthesia (*n* = 3) or i.p. in an awake state (*n* = 3) at rest. Immediately after injection, a multi-frame single-photon emission computed tomography/computed tomography (SPECT/CT) acquisition was initiated with six subsequent time frames of 10 min each. Reconstructed images of the different protocols were assessed and compared by visual analysis by experts and by time-activity-curves generated from regions-of-interest for various organs (normalized uptake values).

**Results:**

Visually assessing overall image quality, the best image quality was found for MIBI for both intravenous injection protocols, whereas TETRO only had comparable image quality after retroorbital injections. These results were confirmed by quantitative analysis where left ventricular (LV) uptake of MIBI after tail vein injections was found significantly higher for all time points accompanied with a significantly slower washout of 16% for MIBI vs. 33% for TETRO (*p* = 0.009) from 10 to 60 min post injection (PI). Interestingly, LV washout from 10 to 60 min PI was significantly higher for TETRO when applied by tail vein injections when compared to retroorbital injections (22%, *p* = 0.008). However, liver uptake was significant and comparable for both tracers at all time points. Radioactivity concentration in the lungs was negligible for all time points and both tracers.

**Conclusion:**

Intravenous MIBI injection (both tail vein and retroorbital) results in the best image quality for assessing myocardial perfusion of the murine heart by SPECT/CT. TETRO has a comparable image quality only for the retroorbital injection route.

## Background

Myocardial perfusion scintigraphy is a well-established method in clinical diagnostic algorithms to study patients with coronary heart disease, cardiomyopathies, and other heart diseases and provides at the same time, not only information on stress and rest perfusion but also functional parameters (gated) and prognostic information with both high sensitivity and specificity [1,2]. From the three commercially available tracers for single-photon emission computed tomography (SPECT), ^201^Thallium, ^99m^Technetium-2-methoxy-isobutyl-isonitrile (MIBI) and ^99m^Technetium-6,9-bis(2-ethoxyethyl)-3,12-dioxa-6,9 diphosphatetradecane (TETRO), the ^99m^Tc-labeled-isonitriles are the ones most frequently employed for myocardial perfusion scintigraphy, mainly due to the convenience of a kit formulation and in-house labeling, a favorable energy spectrum of ^99m^Tc for myocardial SPECT imaging, and a lower radiation exposure when compared with ^201^Tl [3,4].

New diagnostic and therapeutic strategies for cardiovascular diseases are primarily developed in animal models. Mice are of special interest due to logistic advantages and the possibility for genetic engineering, and therefore, a huge variety of mouse models have been established in the last decades. Myocardial perfusion is one of the key parameters to e.g., determine the area-at-risk in the infarction model (ligature of left anterior descending coronary artery) and to assess therapeutic effects such as after interventional, pharmaceutical, or stem cell therapies. The recent development of dedicated small animal SPECT cameras equipped with multi-pinhole collimators enables to “back-translate” clinical SPECT tracers and protocols to small animals such as mice and rats [5,6]. As shown in various studies the quantitative information provided by these small animal, SPECT systems is sufficiently accurate and reproducible to allow noninvasive assessment of radiotracer biodistribution [7,8].

A few studies have successfully employed small animal SPECT to assess myocardial perfusion in rats and mice in various scenarios [9-15] (see Table 1 for overview). In all cases, tracers were injected in variable doses (from 37 MBq/rat up to 450 MBq/mouse). Also, a broad range of data with respect to the start of the acquisition varying from 2 to 30, 45 and 60 min post-injection (PI) for MIBI and 2 to 15, 30 and 60 min PI for TETRO can be observed. Furthermore, only intravenous protocols in anesthetized animals were applied.

**Table 1:** Overview of published SPECT investigations of myocardial perfusion using MIBI and/or TETRO in mice and rats

**Author**	**Species**	**Tracer**	**Injection route**	**Activity (MBq)**	**Time point**	**Scan length**
Acton PD	Rats	99mTc-MIBI	Tail vein	37	1 h PI	30 min
(2006); [9]						
Constantinesco A	Mice	99mTc-tetrofosmin	Femoral vein	350-450	15-20 min PI	48 Projections with 300 beats/projections
(2005); [12]						
Hatada K	Rats	99mTc-MIBI	Saphenous vein	37,7-48,1	2,30, and 60 min PI	5 min
(2004); [14]		99mTc-tetrofosmin				
Liu Z	Rats	99mTc-MIBI	Jugular vein	111-148	0 PI	List mode
(2010); [13]						
Strydhorst JH	Rats	99mTc-tetrofosmin	Tail vein	78 ± 15	30 min p.i	30 min
(2011); [11]						
Wollenweber T	Mice	99mTc-MIBI	Tail vein	370	45 min PI	30 min
(2010); [10]						

Note the broad range in injected doses and acquisition time points after tracer injection

In summary, to date, SPECT protocols to measure myocardial perfusion in mice have not been analyzed systematically with respect to the choice of the perfusion tracer, the application route and the best imaging time window. Therefore, the purpose of this investigation was to systematically evaluate the applicability of the two clinically available ^99m^Tc-labeled tracers for studying myocardial perfusion in mice by small animal single-photon emission computed tomography/computed tomography (SPECT/CT). Beside the classical intravenous application route through the tail vein, alternative injection routes which would be easy to apply (retroorbital and i.p. injections) or available in awake mice (i.p.) were tested.

## Methods

### Radiopharmaceutical preparation

MIBI (CardioTOP; ROTOP Pharmaka AG, Dresden, Germany) and TETRO (MYOVIEW^TM^, General Electric Company, Fairfield, CT, USA) were supplied in commercially available kits. Each vial was reconstituted with ^99m^Tc-O_4_^-^ according to the manufacturer′s instructions.

### Animals

Imaging experiments were approved by the governmental committee on animal welfare North Rhine Westphalia and were performed in accordance with national animal protection guidelines. We studied adult male C57BL/6 mice (19.5 to 32 g in body weight) with free access to standard mouse chow and tap water.

### Multi-pinhole SPECT/CT image acquisition and reconstruction

For each tracer, four imaging protocols employing different routes of administration were tested as follows: (1) intravenous (i.v.) retroorbital injection into the orbital venous plexus (*n* = 5; 150 μl typical injection volume) as published [16-18], (2) i.v. injection through the tail vein (*n* = 5; 150 μl typical injection volume + 150 μl saline flush), (3) intraperitoneal (i.p.; 150 μl typical injection volume) injection under anesthesia (*n* = 3) and (4) i.p. injection in awake mice (*n* = 3; 150 μl typical injection volume). Mice were injected and scanned under isoflurane/oxygen inhalation anesthesia (1.4% isoflurane, 0.3 l O_2_/min); in group 4 anesthesia was only initiated immediately after injection. All animals were injected with a target dose of 4.0 MBq/g body weight. For the image acquisition, we used a NanoSPECT/CT-Plus preclinical camera (Mediso Medical Imaging Systems, Budapest, Hungary), a 4-head gamma camera equipped with multi-pinhole collimators, and a cone beam CT imaging system. In this study we employed nine pinholes/head with a diameter of 1.0 mm and a field of view of 30 × 16 mm resulting in a spatial resolution of 0.7 mm. Three minutes after injection, the animals were placed on the bed of the scanner and underwent a topographic X-ray scan (side view, 9 s duration) for definition of the field of view (FOV). Thereafter, a multi-frame SPECT acquisition consisting of six subsequent scans of identical bed position was initiated 5 min PI, with a scan time of 10 min for each time frame and a total acquisition time of 60 min. Frames were labeled with their mean acquisition time point, e.g., ‘10 min’ for 5 to 15 min SPECT acquisition. Ten projections/scans and 60 s/projection resulting in a minimum of 100 kcounts/projection (in order to achieve high quality images with high statistics) were acquired. At the end of the SPECT measurement, a CT scan was performed (same FOV as the SPECT scan) (55 kVp; 180 projections/rotation; 500 ms exposure time and 1 mm pitch with constant statistics).

**Figure 1:**
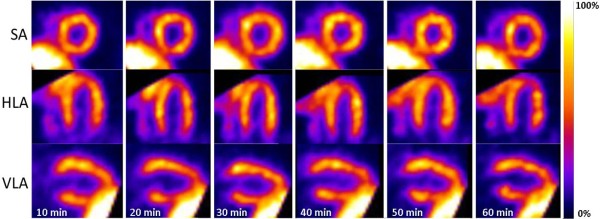
**Characteristic example of a SPECT time series.** Characteristic example of a SPECT time series following an MIBI intravenous tail vein injection showing excellent image quality throughout the entire acquisition time in midventricular short axes (SA), horizontal long axes (HLA) and vertical long axes (VLA).

Images were reconstructed by an ordered-subsets expectation maximization (OSEM) algorithm [19] using the HiSPECT™ (SciVis GMBH, Göttingen, Germany) software. This OSEM algorithm does not include any correction for either attenuation or scatter. Images are not directly reconstructed with units of radioactivity concentration. A conversion to units of radioactivity concentration (bequerels per milliliter) was done with the help of VivoQuant^TM^ (inviCRO, Boston, MA, USA) analysis software (supplied with the camera) intergrading a user-defined calibration factor. Calibrations are made for each radionuclide-aperture combination [5-8]. For reconstruction the same protocol was used for all studies, including a number of nine iterations and one to four subsets as automatically determined by the software. Reconstructed images were resliced into short axis (SA), horizontal long axis (HLA) and vertical long axis (VLA) for visual analysis (Figure 1).

### Visual assessment of image quality

Three experienced readers, blinded to the imaging protocols, independently scored the overall quality of all images (contrast heart to lung and heart to liver, homogeneity, and image statistics/noise) on a 5-point scale as very poor (1), poor (2), moderate (3), good (4) or very good (5); individual results were finally concluded in a consensus reading. Furthermore, the time point with the best image quality was defined for each multi-frame acquisition.

### Quantitative image analysis

Quantitative image analysis was performed using the VivoQuant^TM^ (inviCRO, Boston, MA, USA) analysis software. To derive dynamic radioactivity concentrations in various thoracic tissues, the following volumes of interest (VOIs) were defined on the multi-frame SPECT dataset: three-dimensional segmentations of the left ventricular (LV) myocardium and liver parenchyma were automatically defined on the SPECT images using Otsu thresholding [20,21], subsequently controlled and manually adjusted if needed (e.g., in the inferior wall of the left ventricle, where the liver spillover can be considerable). A standardized blood pool volume of interest (VOI) (0.55 mm^3^) was manually placed in the center of the lumen of the left ventricle. Lung VOIs were derived using thresholding on the reconstructed CT images, and projected onto the SPECT images. Normalized uptake values were calculated from each VOI for each time frame as radioactivity concentration (bequerels per milliliter) corrected for the injected dose per gram body weight (grams per milliliter).

### Statistical analysis

Data are expressed as mean values and standard errors of mean (SEM) and were compared by one way analysis of variance with Bonferroni correction, if applicable. Statistical calculations were performed using IBM SPSS 20, (IBM, Armonk, NY, USA). Significance was inferred at the *p* < 0.05 level.

## Results and discussion

### Visual analysis of SPECT images

**Figure 2:**
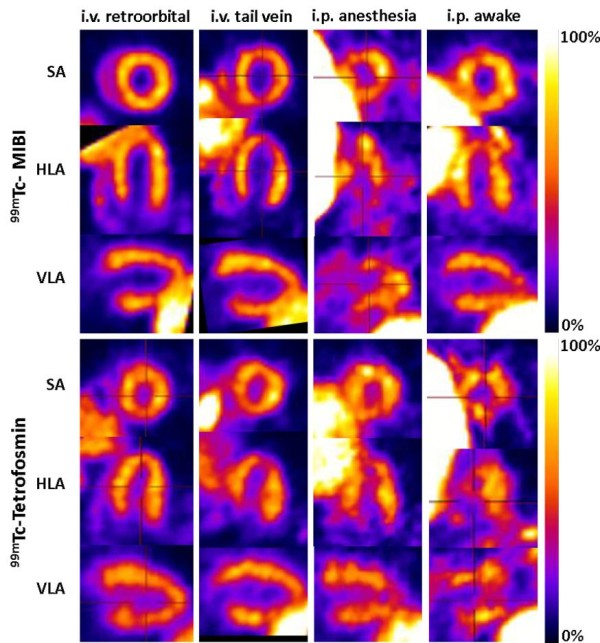
**Examples of the eight different SPECT protocols for the best time frame.** Examples of eight different SPECT protocols for the best time frame in midventricular short axis (SA), horizontal long axis (HLA) and vertical long axis (VLA). This figure demonstrates the good-excellent image quality of the i.v. protocols, whereas the i.p. protocols result in poor and variable image quality.

**Figure 3:**
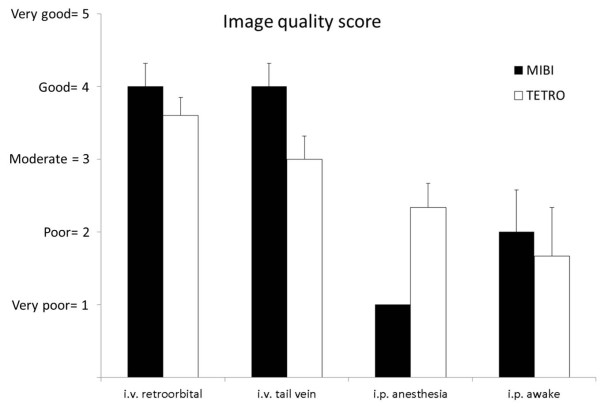
**Results of the consensus reading with respect to the image quality score.** Results of the consensus reading with respect to the image quality score for each of the eight different SPECT protocols (mean image quality score ± SEM). MIBI protocols achieved the highest image quality score for both intravenous injection routes; TETRO was comparable in image quality for retroorbital injections whereas for tail vein injections, image quality of TETRO was scored lower. In contrast to the intravenous protocols, all i.p. protocols revealed very poor image quality.

Characteristic examples of the best imaging time point for each protocol are shown in Figure 2. Results of the blinded consensus reading with respect to image quality for each of the eight different SPECT protocols are summarized in Figure 3. Overall, both tracers achieved images with an adequate contrast between the myocardium and lungs/mediastinum when injected intravenously through the retroorbital plexus or through the tail vein. In contrast to the intravenous protocols, all i.p. protocols revealed very poor and unstable image quality; therefore, these were not further analyzed by quantitative analysis. For the intravenous injection routes, MIBI presented with the best image quality scores for both retroorbital (4.0 ± 0.3) and tail vein (4.0 ± 0.3), whereas TETRO had a slightly lower image quality score of 3.6 ± 0.2 for retroorbital and characteristically lower image quality score of 3.0 ± 0.3 for tail vein injections.

Significant liver uptake was found in most of the protocols potentially hindering the analysis of the apical inferoseptal regions of the LV due to spillover (Figures 1 and 2). The time point for the best image quality was found for MIBI at 48 ± 2 min PI for retroorbital and at 36 ± 5 min PI for tail vein injections, and for TETRO at 30 ± 6 min PI for retroorbital and at 34 ± 5 min PI for tail vein injections.

### Quantitative evaluation

#### Blood uptake

**Figure 4:**
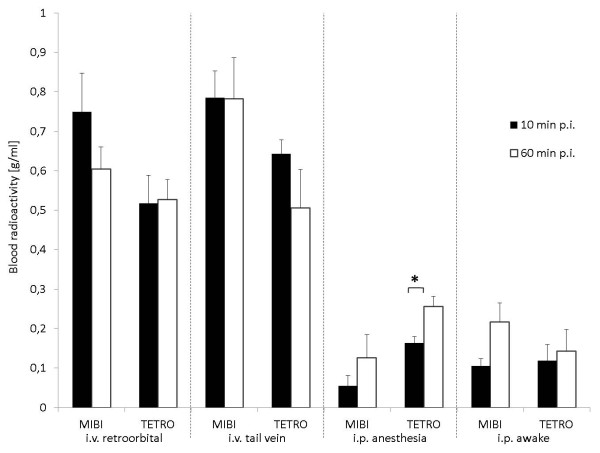
**Blood uptake in the first and last time frame following injection of MIBI or TETRO.** Blood uptake (normalized uptake value ± SEM) in the first (10 min PI; black bars) and last (60 min PI; white bars) time frame following injection of MIBI or TETRO for the four investigated protocols. Statistically different values are marked by an asterisk denoting *p* < 0.05.

Blood radioactivity normalized uptake values were not found to be different between TETRO and MIBI and were stable between 10 and 60 min PI for all intravenous protocols. However, i.p. protocols only showed a fraction of the blood radioactivity measured in intravenous protocols at 10 min with a further increase of blood radioactivity concentration towards the 60 min time frame which reached statistical significance for TETRO in the i.p. anesthesia protocol (Figure 4). This poor and slow uptake from the peritoneum into the blood fits the poor image quality of the i.p. injection protocols.

#### Organ biodistribution

**Figure 5:**
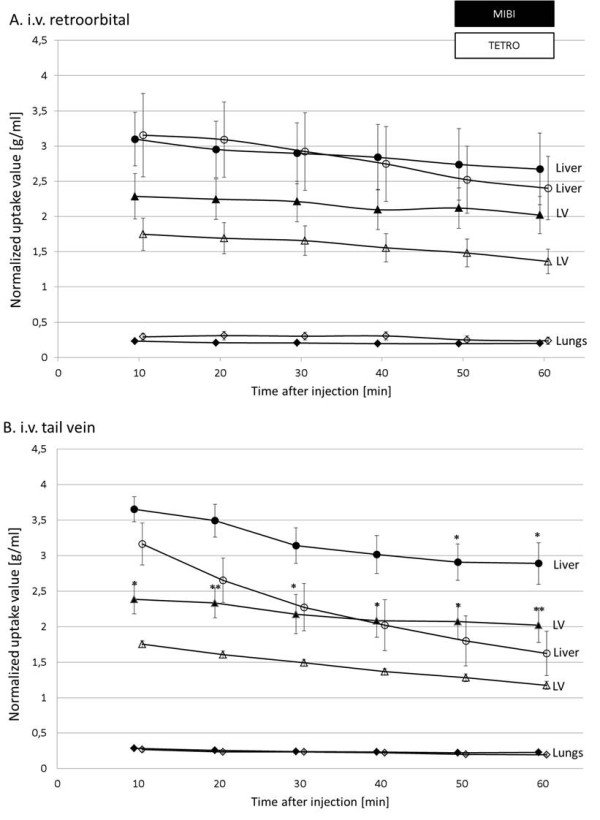
**Uptake (normalized uptake value ± SEM) in various organs in the six time frames following i.v.** retroorbital (**A**) and tail vein injections (**B**) of MIBI (closed symbols) or TETRO (open symbols). Statistically different values are marked by asterisks with single asterisk, *p* < 0.05 and double asterisk, *p* < 0.01. LV, left ventricle.

For each acquisition dynamic radioactivity concentration in different organs such as LV, liver and lungs were measured and corrected for the injected dose per gram body weight by calculation of normalized uptake value for the period between 5 and 65 min PI. Respective time-activity curves are given in Figure 5.

Figure 5A shows retroorbital i.v. injections. Although MIBI showed higher LV uptake throughout all time frames when compared to TETRO, this did not reach statistical significance. With 22% TETRO showed a significantly higher washout from the LV in the time interval from 10 to 60 min when compared to MIBI (11%, *p* = 0.004). Uptake in the lungs was negligible for all time points and both tracers. However, liver uptake was significant and comparable for both tracers at all time points with both tracers showing a slight liver washout (MIBI, 16% vs. TETRO, 23%, *p* = 0.48) in the imaging interval.

Figure 5B shows tail vein i.v. injections. (In contrast to retroorbital injections, LV uptake of MIBI after tail vein injections was significantly higher for all time points accompanied with a significantly slower washout of 16% for MIBI as compared to 33% (*p* = 0.009) for TETRO from 10 to 60 min PI.

Interestingly, LV washout from 10 to 60 min PI was significantly higher for TETRO when applied by tail vein injections (33%) when compared to retroorbital injections (22%, *p* = 0.008). Radioactivity concentrations for MIBI and TETRO in the lungs were comparable to the retroorbital injection case. However, liver uptake of MIBI and TETRO were similar early after injection but significantly lower for TETRO from the 50 min time point on.

This systematic evaluation investigated for the first time various protocols for clinically available perfusion tracers, MIBI and TETRO, to study myocardial perfusion in mice using small animal SPECT/CT. We conclude that intravenous MIBI injections are resulting in the best image quality. TETRO has a comparable image quality for retroorbital injections, however, when combined with tail vein injections TETRO shows inferior image quality as compared to MIBI.

In contrast to intravenous injections, i.p. injection protocols resulted in poor and variable image quality. This could be explained by unstable and undefined resorption, thus resulting in unpredictable appearance and kinetics in the animals' circulation. I.p. injections would have been attractive alternatives to the i.v. injections due to the simple injection technique which is also available in the awake mice, thus potentially better allowing imaging of the mouse heart physiology as compared to the anesthesia situation.

It is well known that MIBI and TETRO are rapidly and efficiently extracted from the blood by cardiac myocytes. In the murine heart we demonstrate a major difference of the left ventricular uptake in favor of MIBI at all time points when using tail vein injections. Although all SPECT/CT measurements in mice were performed at a resting state, the resting myocardial blood flow in mice under isoflurane anesthesia is in the range of 7 to 17 ml/min/g, which is already higher than the stress myocardial blood flow in patients (2 to 4 ml/min/g) [22,23]. In such high-flow situations in patients, the “roll-off phenomenon” (nonlinear accumulation of radioactivity with increasing blood flow) has been observed to be significantly lower for MIBI than for TETRO [24].

In addition to the myocardial uptake, our study also confirms data from human studies demonstrating substantial differences in biokinetics, such as a faster hepatic clearance of TETRO in comparison to MIBI, thus resulting in an early improvement of the heart/liver ratio, potentially making TETRO more suitable for early imaging than MIBI [25-27]. In line with this hypothesis, the best image quality for retroorbital injections was found earlier for TETRO (30 min PI) than for MIBI (48 min PI) in the visual analysis.

Surprisingly, the intravenous application routes, retroorbital vs. tail vein, had a significant impact on the biodistribution in the case of TETRO but not for MIBI: the washout from the myocardium was significantly higher after tail vein injection when compared to the retroorbital injection for TETRO. This could be explained by a prolonged washout of TETRO from the retroorbital venous plexus resulting in a prolonged concentration of TETRO in the blood based on physicochemical property differences between TETRO and MIBI.

As to date, only a few groups have published data on myocardial perfusion measured by SPECT in small animals such as rats and mice (Table 1). In all cases tracers were injected in variable doses (from 37 MBq/rat up to 450 MBq/mouse). Also a broad range of data with respect to the start of the acquisition varying from 2 to 30, 45 and 60 min PI for MIBI and 2 to 15, 30 and 60 min PI for TETRO can be observed. In our study, we show that a short acquisition time of 10 min following an injection of a reasonable radioactivity dose of 4 MBq/g mouse results in a good-excellent image quality. For both tracers, the best imaging time window was found between 30 and 50 min PI allowing fast imaging algorithms.

##### Study limitations

After the analysis of three imaging studies per i.p. group, we decided not to further investigate these protocols for reasons of good animal practice. Because of the previously described roll-off phenomenon under high-flow conditions, which in mice are already found in resting conditions, we did not aim for absolute quantification of myocardial perfusion in this study. As the focus of this study was on the assessment of different application routes and tracers, no model of myocardial pathology (ischemia/infarct) was used. We therefore cannot finally conclude about the potential tracer specific differences for the detection of perfusion defects.

## Conclusions

Intravenous MIBI injection (both tail vein and retroorbital) results in the best image quality for assessing myocardial perfusion of the murine heart by SPECT/CT with the best acquisition time point 40 min PI. TETRO has a comparable image quality only for the retroorbital injection route with the best imaging time point at 30 min PI.

## Abbreviations

FOV, field of view; i.v., intravenous; i.p., intraperitoneal; LV, left ventricle; MIBI, 99mTc-sestamibi; PI, post injection; SPECT, single photon emission computed tomography; TETRO, 99mTc-Tetrofosmin; VOI, volume of interest.

## Competing interests

DM is a consultant of Mediso Ltd. All other authors declare that they have no competing interests.

## Authors’ contributions

AV has made substantial contributions to conception and design, acquisition, analysis and interpretation of data and drafted the manuscript. SH participated in the design and interpretation of the study and has critically revised the manuscript. DM assisted in the design of the imaging protocol and data analysis. OS has inspired the question of translation from mice to man and has critically revised the manuscript. MS conceived the study, participated in its interpretation and has critically revised the manuscript. All authors read and approved the final manuscript.
